# Impact of interventions to prevent and manage preeclampsia and eclampsia on stillbirths

**DOI:** 10.1186/1471-2458-11-S3-S6

**Published:** 2011-04-13

**Authors:** Mehnaz Jabeen, Mohammad Yawar Yakoob, Aamer Imdad, Zulfiqar A Bhutta

**Affiliations:** 1 Division of Women & Child Health; The Aga Khan University, Stadium Road, P.O. Box 3500, Karachi-74800, Pakistan

## Abstract

**Background:**

Pre-eclampsia and Eclampsia are relatively common complications of pregnancy, leading to considerable maternal and fetal mortality and morbidity. We sought to review the effect of aspirin, calcium supplementation, antihypertensive agents and magnesium sulphate on risk stillbirths.

**Methods:**

A systematic literature search was conducted to identify studies evaluating the above interventions. We used a standardized abstraction and grading format and performed meta-analyses where data were available from more than one studies. The estimated effect on stillbirths was determined by applying the standard Child Health Epidemiology Reference Group (CHERG) rules for multiple outcomes. For interventions with insufficient evidence for overall effect, a Delphi process was undertaken to estimate effectiveness.

**Results:**

We identified 82 relevant studies. For aspirin, maganesium sulphate and use of antihypertensive we found an insignificant decrease in stillbirth and perinatal mortality. For calcium supplementation, there was a borderline significant reduction in stillbirths (RR 0.81, 95 % CI 0.63-1.03). We undertook a Delphi consultation among experts to assess the potential impact of a package of interventions for the management of pre-eclampsia and eclampsia (antihypertensive, magnesium sulphate and C-section if needed). The Delphi process suggested 20% reduction each in both antepartum and intrapartum stillbirths with the use of this package.

**Conclusions:**

Despite promising benefits of calcium supplementation and aspirin use cases on maternal morbidity and eclampsia in high risk cases, further work is needed to ascertain their benefits in relation to stillbirths. The Delphi process undertaken for assessing potential impact of a package of interventions indicated that this could be associated with 20% reduction in stillbirths, for input into LiST.

## Background

Hypertensive disease of pregnancy (HDP) is one of the most common complications of pregnancy, occurring in 5-8% of the pregnancies [1]. Hypertensive disorders are the second most common cause of maternal deaths worldwide [2]. There are several major categories of hypertensive disorders in pregnancy ranging from mild to moderate rise in blood pressure without proteinuria [usually called pregnancy induced hypertension (PIH)], preeclampsia (hypertension with proteinuria), severe preeclampsia and eclampsia [3]. Maternal hypertension, even of the mild to moderate category, can lead to adverse perinatal outcomes like low birth weight, prematurity, stillbirth and intrauterine growth retardation [4]. A population-based study of 57 million singleton live- and stillbirths has shown that pregnancy induced hypertension (PIH) is associated with increased risk of stillbirth and neonatal mortality [5]. The risk of stillbirth was higher in women having their second or higher order births [(Odds Ratio (OR) =2.24, 95% confidence interval (CI) =2.11-2.37)] compared with women having their first birth (OR=1.52, 95% CI=1.40-1.64).

Detection and prevention of maternal hypertensive disorders is important in order to avoid morbidity and mortality associated with them [6]. Potential interventions for reducing the risk of preeclampsia include calcium and aspirin used for prevention and use of anti-hypertensive drugs and magnesium sulphate for management of preeclampsia/eclampsia. A 17% reduction in the risk of preeclampsia was noted with the use of antiplatelet agents – mostly low dose aspirin - in pregnant women at risk of preeclampsia (RR 0.83 95% CI 0.77-0.89) [7]. A Cochrane review [8] that included 13 randomized trials comprising 15,730 women has shown that calcium supplementation during pregnancy reduces the incidence of preeclampsia by 55 % [Relative risk (RR) =0.45 95 % confidence interval (CI) 0.31-0.65] and that of gestational hypertension by 35 % [RR=0.65, 95 % CI 0.53-0.81]. The reduction in the risk of preeclampsia was greatest for women at high risk of developing preeclampsia (RR 0.22, 95% CI 0.12-0.42), and for those with low baseline intake of calcium (RR 0.36, 95% CI 0.20-0.65). A variety of antihypertensive agents including beta-blockers like labetalol [9,10], atenolol [11,12], oxeprenolol [13]; calcium-channel blockers like isradipine[14], nifedipine [15,16]; and other antihypertensive drugs like methyldopa (an alpha-blocker) [17-19] have been shown to be effective in controlling blood pressure during pregnancy. Magnesium sulphate (MgSO4) supplementation has been shown to be effective for prevention of eclampsia in pre-eclamptic women [20]. A Cochrane review [21] that included six trials for MgSO4 analysis has shown that compared with placebo or no anticonvulsant, MgSO4 reduced the risk of eclampsia by 59 % [RR 0.41, 95% CI 0.29 to 0.58].

The purpose of this paper was to review the effect of calcium supplementation, aspirin, antihypertensive agents, and magnesium sulphate on stillbirths and perinatal mortality related to maternal hypertensive disorders. This paper is a part of series of papers for the Lives Saved Tool (LiST). The process of generating a point estimate for efficacy of an intervention involves qualitative evaluation of available evidence according to Grading of Recommendations, Assessment, Development and Evaluation (GRADE) criteria [22] and quantitative measure according to Child Health Epidemiology Reference Group (CHERG) rules [23]. For more details of the review methods, the adapted GRADE approach or the LiST model see the methods paper [23] and other articles in this supplement.

## Methods

We systematically reviewed all published literature from 1957 to June 2010 to identify studies that reported the effect of calcium supplements, aspirin, antihypertensive drugs or magnesium sulphate on stillbirths and perinatal mortality as outcomes amongst pregnant women. As per CHERG guidelines [23], we searched PubMed, the Cochrane Library and all WHO regional databases and included publications available in any language [23].

Two search strategies were used as follows:

1) (aspirin OR antiplatelet OR antihypertensive* OR "beta-blocker*" OR "calcium channel blocker*" OR "alpha-blocker*" OR "magnesium sulphate") AND (pregnancy or maternal) AND (stillbirth* OR “perinatal mortality” OR “fetal death*" OR eclampsia OR "severe preeclampsia" OR “intrauterine death” OR “preeclampsia” OR” preeclampsia*).

2) “Calcium” AND “pregnancy” AND (“Hypertension” OR “preeclampsia” OR “blood pressure” OR “Stillbirth” OR “Intrauterine death”).

Every effort was made to gather unpublished data when reports were available for full abstraction. Previous reviews were also hand-searched for relevant studies not picked up in the primary literature search [3,24-26]

### Inclusion/exclusion criteria

We included randomized and quasi-randomized controlled trials that evaluated the following as an intervention: calcium supplements, aspirin with or without other antiplatelet agents, antihypertensive agents of any category, and magnesium sulphate. Studies were included if they had an identical control group that was given either placebo or no treatment. The studies were selected irrespective of the dose used. For calcium, only those studies were included in which baseline calcium intake of participants was low as calcium supplementation during pregnancy is effective only in population with low calcium intake. We excluded any studies reporting only before-after comparisons as well as observational studies and in addition we excluded those studies that did not report outcomes of interest for this review.

### Abstraction, analysis, and summary measures

All studies that met the final inclusion and exclusion criteria were double-data abstracted into a standardized form for each outcome. We extracted the data for study design; location; population; methods; limitations; assessment of blinding, allocation concealment; and outcomes of interest. Each study was assessed and graded according to the CHERG adaptation of the GRADE technique. Each study was assigned a final quality grade of “high” “moderate” “low” or “very low” on the basis of strengths and limitations of study [22,23]. Studies receiving a grade of ‘very low’ were excluded from the analysis. The grading of overall (pooled) evidence was based on three components: (1) the volume and consistency of the evidence; (2) the size of the pooled effect and (3) the strength of the statistical evidence reflected in the p-value [23]. A similar grading of ‘high’ ‘moderate’ ‘low’ and ‘very low’ was used for grading the overall evidence indicating the strength of an effect of the intervention on specific health outcome [23]

We undertook meta-analyses where data were available from more than one study for an outcome and reported Mantel-Haenszel pooled relative risks and corresponding 95 % confidence intervals. The primary outcome of interest was stillbirth. Statistical heterogeneity in the pooled data was assessed by visual inspection of overlap of confidence intervals and P-value (P < 0.10 was taken to mean substantial heterogeneity). The assessment of statistical heterogeneity in the pooled analysis was done by visual inspection i.e. the overlap of the confidence intervals among the studies, Chi square (P-value) of heterogeneity in the meta-analyses and I^2^ statistics. A low P value (less than 0.10) or a large chi-squared statistics relative to its degree of freedom and I^2^ values greater than 50% were taken as substantial and high heterogeneity. In situations of substantial or high heterogeneity being present, causes were explored by sensitivity analysis and random effects model were used.

## Results

**Figure 1:**
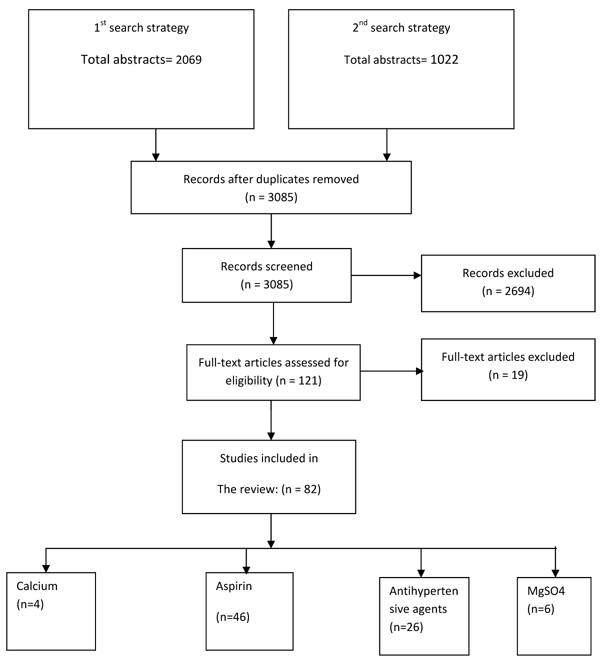
Flow chart for study selection

We identified 2069 titles from the first search strategy and 1022 titles from the second, in searches conducted in databases including PubMed and the Cochrane Library (Figure 1). After initial screening of these titles and abstracts, we reviewed 121 papers to identify outcomes of interest and included 82 papers in the final database. All the included studies were blinded, randomized, controlled treatment trials. The detailed data extraction with the limitation of studies is shown in Additional File 1. In tables 1, 2, 3, 4, we report the quality assessment of studies of each intervention by outcome, as well as results from corresponding meta-analyses.

**Table 1:** Quality assessment of trials of aspirin alone in high-risk pregnancies for prevention of preeclampsia

	Quality Assessment					Summary of Findings		
				**Directness**		**No of events**		

**No of studies (ref)**	**Design**	**Limitations**	**Consistency**	**Generalizability to population of interest**	**Generalizability to intervention of interest**	**Intervention**	**Control**	**Relative Risk (95% CI)**

***Stillbirths: LOW outcome specific quality***								

21	RCT	High non-compliance rate, Small sample size, Placebo not used, Not double-blinded.	Consistent; Low heterogeneity (p=0.62); Results statistically insignificant	1 study in developing country	Yes	113	95	1.15 (0.88, 1.49)

***Perinatal mortality: MODERATE/LOW outcome specific quality***								

13	RCT	Low compliance; Small sample size	Consistent; Low heterogeneity (p=0.39); Results statistically insignificant	3 studies in developing countries	Yes	187	209	0.89 (0.74, 1.08)

**Table 2:** Quality assessment of studies of calcium supplementation during pregnancy to prevent stillbirths from maternal hypertensive disorders:

Quality Assessment				Directness		Summary of findings		
**No. of studies (Ref)**	**Design**	**Limitations**	**Consistency**	**Generalizability to Population of Interest**	**Generalizability to intervention of Interest**	**Events in intervention group**	**Events in control group**	**Relative Risk ( 95 % CI)**

**Stillbirth: Moderate outcome specific quality**								

3	RCT	None	Consistent (I^2^ =0)	Yes all from developing countries	Dose of calcium supplementation ranged from 1.5-2 g/day in all the included studies	117	145	0.81 (0.63-1.03)a

**Perinatal mortality: Low outcome specific quality**								

4	RCTs	None	Consistent (I^2^ =0)	Yes All from developing countries	Dose of calcium supplementation ranged from 1.5-2 g/day in all the included studies	154	179	0.86 (0.70-1.07)a

**Table 3:** Quality assessment of trials of antihypertensive agents in pregnancies with mild to moderate hypertension

	Quality Assessment					Summary of Findings		
				**Directness**		**No of events**		

**No of studies (ref)**	**Design**	**Limitations**	**Consistency**	**Generalizability to population of interest**	**Generalizability to intervention of interest**	**Intervention**	**Control**	**Relative Risk (95% CI)**

***Stillbirths: MODERATE/LOW outcome specific quality***								

18	RCT	Small sample size, Placebo not used	Consistent; Low heterogeneity (p=0.93); Results statistically insignificant	3 studies in developing countries	Yes	18	16	1.14 (0.60, 2.17)

***Perinatal mortality: MODERATE/LOW outcome specific quality***								

20	RCT	Small sample size, Placebo not used	Consistent; Low heterogeneity (p=0.97); Results statistically insignificant	3 studies in developing countries	Yes	30	82	0.96 (0.60, 1.54)

**Table 4:** Quality assessment of trials of magnesium sulphate in pregnancies with preeclampsia

	Quality Assessment					Summary of Findings		
				**Directness**		**No of events**		

**No of studies (ref)**	**Design**	**Limitations**	**Consistency**	**Generalizability to population of interest**	**Generalizability to intervention of interest**	**Intervention**	**Control**	**Relative Risk (95% CI)**

***Stillbirths: HIGH/MODERATE outcome specific quality***								

3	RCT	None	Consistent; Low heterogeneity (p=0.34); Results statistically insignificant	2 studies in developing countries	Yes	424	426	0.99 (0.87, 1.12)

***Perinatal mortality: HIGH/MODERATE outcome specific quality***								

2	RCT	None	Consistent; Low heterogeneity (p=0.45); Results statistically insignificant	1 study in developing country	Yes	538	541	0.98 (0.88, 1.10)

### Aspirin in high-risk pregnancy

**Figure 2:**
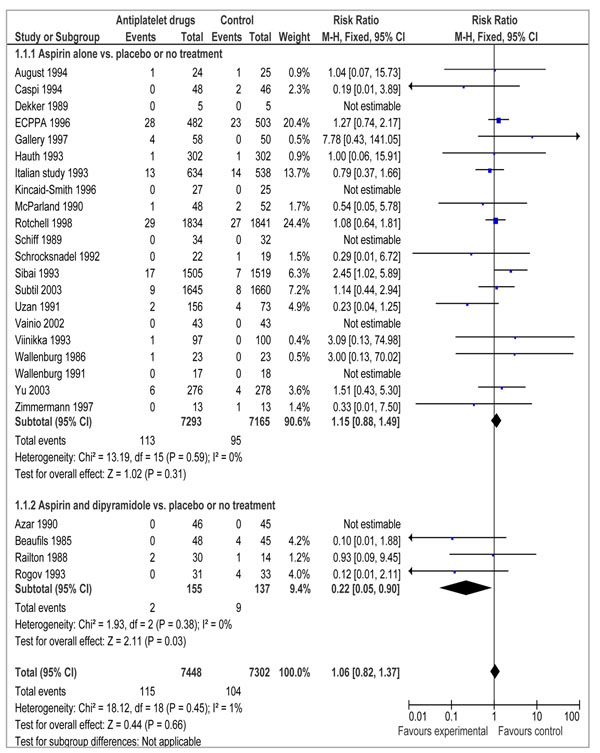
Forest plot for the effect of aspirin on stillbirths

To estimate the effect of aspirin in high-risk pregnancies for prevention of preeclampsia, we found 21 studies which reported data on the effect of aspirin alone on stillbirths [1,4,9,12,13,15-19,27-37] and 3 studies reporting combined effects of aspirin and diypyramidole on stillbirths [38-40]. A total of 13 studies reported perinatal mortality [1,4,15,17,27,29,31,33,35,36,39,41-43] (Table 1). The effect of aspirin alone showed no significant effect on risk of stillbirths (RR= 1.15; 95% CI: 0.88 to 1.49) (Figure 2). Combining studies of aspirin with dipyramidole with aspirin alone yielded similar results (RR= 1.06; 95% CI: 0.82 to 1.37).

### Calcium supplementation in low-intake population

**Figure 3:**
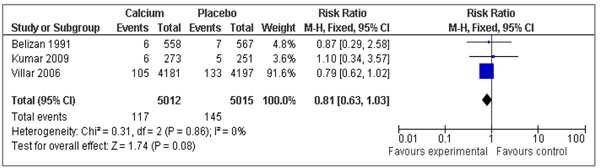
Forest plot for the effect of calcium supplementation on stillbirths

To estimate the effect of calcium supplementation in pregnant women in low-intake population, we found 3 studies which reported data on stillbirths, [55-57] and 4 studies which reported perinatal mortality (Table 2). Our analysis suggested that calcium supplementation during pregnancy could potentially reduce stillbirths by 19%, however results were not statistically significant (RR 0.81, 95 % CI 0.63-1.03) (Figure 3). These data suggest the need for further large scale studies of calcium supplementation in populations with specific assessment of stillbirth outcomes.

### Antihypertensive agents in pregnancy with mild to moderate hypertension

**Figure 4:**
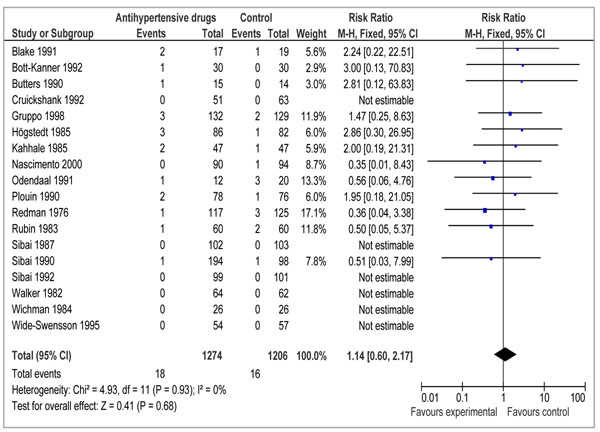
Forest plot for the effect of antihypertensive agents on stillbirths

To estimate the effect of antihypertensive agents in pregnant women with mild to moderate hypertension, we found 18 studies which reported data on stillbirths [10,14,58-73] and twenty studies which reported data on perinatal mortality [14,58,60,61,63-66,68,69,71-80] (Table 3).The effect of antihypertensive drugs showed no effect for stillbirths (RR= 1.14; 95% CI: 0.60 to 2.17) (Figure 4).

### Magnesium sulphate in pregnancy with preeclampsia

**Figure 5:**
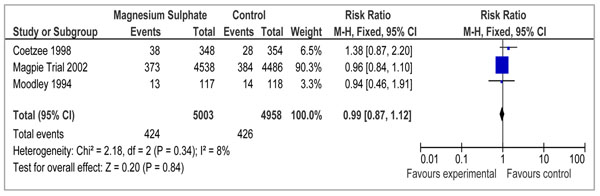
Forest plot for the effect of magnesium sulphate on stillbirths

To estimate the effect of magnesium sulphate for pregnancies with preeclampsia versus placebo or no treatment, we found 3 studies which reported data on stillbirths[82-84], 2 studies which reported perinatal mortality [82,83] (Table 4). There was no impact of use of magnesium sulphate on stillbirths (RR= 0.99; 95% CI: 0.87 to 1.12) (Figure 5).

### Delphi process for establishing expert consensus

**Figure 6:**
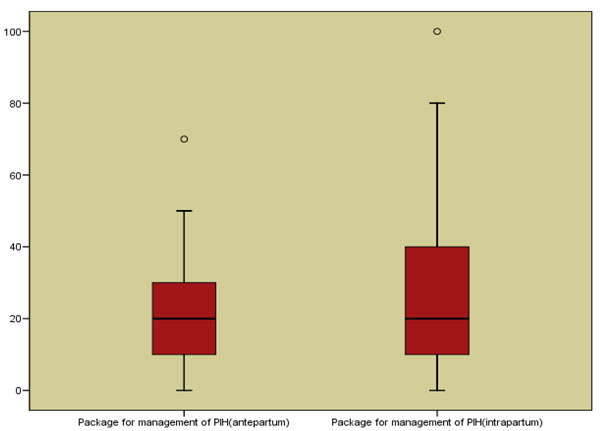
Box plot for the Delphi results for the HDP package

As there was no convincing evidence in favor or against of use of antihypertensive and magnesium sulphate with respect to stillbirths, we sought expert consensus via the Delphi method [23]. The panel invited to participate were experts belonging to six WHO regions (South Asia, Africa, Western Europe, Eastern Europe, North America, and Australia) and a range of disciplines including maternal health, public health, obstetrics/gynecology, and midwifery. 33 experts agreed to participate in the Delphi process. The questionnaire was developed by MYY and ZAB, and refined after several rounds of pilot testing. The questionnaire was sent by email and included the background and aims of the Delphi and estimates of effect that were available from the literature for different scenarios. The median response and range were determined for each question. Consensus was defined a priori as an interquartile range in responses of not more than 30% for each question and in this instance; consensus was achieved after first iteration. The Delphi consensus suggested median effect of 20% reduction in antepartum and intrapartum stillbirths with the HDP package with interquartile ranges of 10-30% and 10-40% respectively (Figure 6).

## Discussion

Over 2.5 million babies are born dead each year, making stillbirth one of the most important adverse outcomes of pregnancy[88]. Hypertension during pregnancy is a risk factor for stillbirth [89-91] especially in low socioeconomic settings [92]. The two most commonly used preventive measures are; use of aspirin in high risk pregnancies and calcium supplementation in populations with low calcium intake [20]. In this review we have found that use of aspirin was not associated with a decrease in stillbirths (RR= 1.15; 95% CI: 0.88 to 1.49). Results of this review are different from that of Cochrane review [25] on the topic (aspirin) as data for stillbirth were pooled with miscarriages in the Cochrane review. The combined results in the Cochrane review also showed no effect of aspirin (RR=0.96; CI= 0.78 to 1.18]. In any case, despite having a protective effect against pre-eclampsia, there was no convincing evidence to recommend use of ‘aspirin alone’ for reduction of stillbirths for input to the Live Saved Tool (LiST).

Analysis in this review has shown that calcium supplementation during pregnancy reduced stillbirths by 19 %; however results were not statistically significant (RR 0.81, 95 % CI 0.63-1.03). A Cochrane review on effectiveness of calcium supplementation during pregnancy [24] also showed a statistically insignificant 30% reduction in the outcome of stillbirths or death before discharge (RR 0.70, 95% CI 0.69 to 1.09). There was however 55 % reduction in risk of preeclampsia (RR 0.45, 95% CI 0.31 to 0.65). Although the evidence in support of calcium supplementation for women at risk of low intake is robust [24], further research is needed with assessment of the full range of pregnancy outcomes before this can be recommended for inclusion in the LiST model for prevention of stillbirth.

This review has shown that use of antihypertensive drugs has no effect on incidence of stillbirths, which is similar to the results from the Cochrane review (RR=1.14 CI=0.60 to 2.17) [3].

It should be noted that most data included in this review for aspirin, antihypertensive agents and magnesium sulphate was from developed countries, as data from developing countries was scarce. Several studies had too small a sample size to be able to detect differences in stillbirths. Moreover, noncompliance rates to the aspirin were as high as 20% in certain studies. In a few studies, a no-treatment group was used as a control group instead of a placebo group, which may have affected the results.

Given these limitations of the data, we also used the Delphi process to generate an impact estimate for impact of a package of interventions that involve detection and management of hypertensive disease of pregnancy. The package included use of an appropriate antihypertensive, magnesium supplementation in case of preeclampsia/eclampsia and availability of C-section when required. The selection of this package was based on the fact these interventions are accepted standards of care for management of preeclampsia/eclampsia[6]; however their role in prevention of stillbirths has not been extensively studied as described above. We therefore contacted experts in the field (total 33) and asked their opinion on how effective this package of intervention could be to reduce incidence of stillbirths. The results yielded a reduction of 20 % in antepartum and intrapartum stillbirths. It should be noted that this evidence is based on expert’s opinion only and considered as the weakest evidence in the CHERG’s methods of evaluation of effectiveness of an intervention [23]. We therefore recommend that future research should focus on better understanding of the role of detection and management of gestational hypertensive disorders in prevention of stillbirths. It is possible that a package of interventions has an effect that is greater than the individual components and the inclusion of, for example, C Section, may explain the effect attributed to the package as a whole by the experts. Finally, it is possible the experts were unaware of negative summary data of components of the package.

## Conclusion

In conclusion, aspirin and calcium play a significant role in prevention of preeclampsia however their role in reducing stillbirths is not well established. Antihypertensive and magnesium sulphate supplementation for hypertensive disorders in pregnancy reduce morbidity and mortality associated with these disorders however their role in reducing stillbirths is not clear. Based on expert’s opinion a package of detection and management of gestational hypertensive disorders (Antihypertensive, MgSO4 and C-Section) was estimated to reduce the incidence of stillbirths by 20 %.

## Competing interests

The authors declare no conflict of interest

## Authors’ contributions

Professor Zulfiqar A Bhutta developed the review parameters and secured support. Drs  Mehnaz  Jabeen, Yawar Yakoob and Aamer Imdad undertook the literature search, data extraction and analysis under the supervision of Professor Bhutta. Professor Bhutta gave advice in all the aspects of the project and was the overall supervisor.

## Supplementary Material

Additional File 1 Data extraction sheet for studies included in the review Click here for file
